# Supplementary material for: The effects of endogenous FSH and its receptor on oogenesis and folliculogenesis in female Alligator sinensis
Source: BMC Zool. 2023 Jul 4;8:8. doi: 10.1186/s40850-023-00170-z (PMC10318752; doi:10.1186/s40850-023-00170-z)
Supplement: Supplementary file 1 — Supplementary Material 1 [file 40850_2023_170_MOESM1_ESM.docx]

**The effects of endogenous FSH and its receptor on oogenesis and folliculogenesis in female *Alligator sinensis***

Haitao Nie^1^, Yunlu Xu^1^, Yuqian Zhang^1^, Yue Wen^1^, Jixiang Zhan^1^, Yong Xia^1^, Yongkang Zhou^2^, Renping Wang^2^, Xiaobing Wu^1*^

*^1^ Anhui Provincial Key Laboratory of the Conservation and Exploitation of Biological Resources, College of Life Sciences, Anhui Normal University, Wuhu, Anhui Province, 241000, People's Republic of China*

*^2^ Alligator Research Center of Anhui Province, Xuanzhou 242000, People's Republic of China*

^*^ Corresponding Author: Xiaobing Wu, E-mail address: [wuxb@ahnu.edu](mailto:wuxb@mail.ahnu.edu).cn

**ABSTRACT**

**Background:** The precise mechanisms of hormone action responsible for the full course of events modulating folliculogenesis in crocodilian have not been determined, although histological features have been identified.

**Results:** The *Alligator sinensis* ovarian morphological characteristics observed at 1, 15, 30, 60, 90, and 300 days post hatching(dph) revealed that the dynamic changes in germ cells varied in different meiotic and developmental stages, confirming that the processes of folliculogenesis were protracted and asynchronous. The presence of endogenous follicle-stimulating hormone(FSH) mRNA and protein expression within the cerebrum at 1 dph, in parallel with the increase in germ cells within the germ cell nests(Nest) from 1 dph to 15 dph, suggested that endocrine regulation of the pituitary-gonad axis is an early event in oogonia division. Furthermore, the endogenous expression of FSH showed a trend of negative feedback augmentation accompanied by the exhaustion of maternal yolk E_2_ observed at 15 dph. Such significant elevation of endogenous FSH levels was observed to be related to pivotal events in the transition from mitosis to meiosis, as reflected by the proportion of oogonia during premeiosis interphase, with endogenous FSH levels reaching a peak at the earliest time step of 1 dph. In addition, the simultaneous upregulation of premeiotic marker *STRA8* mRNA expression and the increase in endogenous FSH further verified the above speculation. The strongly FSHr-positive label in the oocytes within Pre-previtellogenic follicles was synchronized with the significant elevation of ovarian cAMP detected at 300 dph, which suggested that diplotene arrest maintenance during early vitellogenesis might be FSH dependent. In addition, preferential selection in asynchronous meiotic initiation has been supposed to act on somatic supportive cells and not directly on germ cells via regulation of FSH that in turn affects downstream estrogen levels. This suggestion was verified by the reciprocal stimulating effect of FSH and E_2_ on the accelerated meiotic marker *SYCP3* and by the inhibited cell apoptosis demonstrated in ovarian cell culture in vitro.

**Conclusion:** The corresponding results contribute an expansion of the understanding of physiological processes and shed some light on the specific factors responsible for gonadotropin function in the early folliculogenesis of crocodilians.

**Keywords**: Crocodilian; Estrogen signaling; Folliculogenesis; Follicle-stimulating hormones; Follicle-stimulating hormone receptor

**Background**

The potential fecundity of females of a given vertebrate species is ultimately dependent on an adequate supply of oogonia produced by mitotic proliferation, accompanied by the primary oocytes invested with granulosa cells to form primordial follicles. Mechanistic elucidation of early germ cell differentiation during oogenesis and folliculogenesis in vertebrates is fundamental to advancing our knowledge of germline development. In each of these organisms, most of the single oogonia divide synchronously via intercellular bridges with incomplete cytokinesis to form a cluster of cells named the germ cell nest [[1](#_ENREF_1)]. As the building blocks of the germline cyst, mitotically active germ cells progress through the stages of meiotic prophase, such as the leptotene, zygotene, pachytene stages, and arrest in the diplotene stage [[2](#_ENREF_2)]. A widespread belief in reproductive biology is that in most mammals, female germline stem cells are transformed into primordial follicles (Pmfs) during embryogenesis and cease after birth [[3](#_ENREF_3)]. However, the entrance of oogonia into meiotic prophase has been observed throughout the reproductive lifetime of reptiles, even though the nature of factors that initiate and regulate oogenesis is still poorly understood[[4](#_ENREF_4)]. According to an excellent comparative review of gametogenesis in reptiles and birds, the loss of the continual presence of oogonia in the adult ovary appears to have evolved to reduce the energy expended during egg production [[5](#_ENREF_5)]. As crocodilians are the organisms that are phylogenetically most closely related to birds, knowledge of the developmental biology of their oogenesis and folliculogenesis could provide answers concerning the evolution of archosaurian oviparity. Although a large number of well-studied ovarian morphological developments varying from embryonic [[6](#_ENREF_6)] to neonatal [[7](#_ENREF_7), [8](#_ENREF_8)] to adult individuals [[9](#_ENREF_9)] have been observed, the possible cellular and molecular mechanisms are less known in crocodile species than in other vertebrates such as fish, amphibians and mammals.

The gonadotropin follicle-stimulating hormone (FSH) is a glycoprotein that plays a central role in mammalian reproduction and development. Similarly, various studies in reptiles have provided circumstantial evidence that FSH has a mediating effect on oogonial multiplication. For instance, the oogonial division of intact juvenile *Malaclemys terrapin* significantly increased following the administration of mammalian ovine pituitary material [[10](#_ENREF_10)]. Similarly, treatment with mammalian FSH significantly increased [^3^H]-thymidine-labeled oogonia in adult hypophysectomized *Anolis. carolinensis* [[11](#_ENREF_11)]. Regarding crocodilians, the alkaline extract from sheep pituitaries increases the number of oogonia with mitotic activity in the immature *Alligator mississippiensis* [[12](#_ENREF_12)]. However, it must be cautioned that although hypophysectomy treatment seems to be the ideal model, it disturbs the entire hormonal milieu, and it is not clear whether other endocrine factors in the absence of gonadotropins influence the mitotic activity of oogonia. Additionally, although extensive work has been carried out to understand the steroid signaling associated with nest breakdown in placental amniotes, the body of literature includes studies of other species, such as oviparous amniotes. For oviparous amniotic animals, maternally derived steroid substrates are suggested to serve as a primary reservoir of steroid substrates directing the early embryonic development of oviparous reptiles, including crocodiles. Such maternal yolk is found to be present in both the embryonic stage and the early stage of hatching, which has been confirmed to be the crucial time node of gametogenesis, such as oocyte proliferation and differentiation [[13](#_ENREF_13)]. However, the relationship between maternally derived steroid substrates and progeny female gametogenesis has not been investigated. It is not clear when the connection between maternal and endogenous steroid hormone signaling is extended for the duration of oogenesis and folliculogenesis.

In the present research, dynamic changes in the number of germ cells, including oogonia, primordial follicles (Pmf), primary follicles (PF) and previtellogenic follicles (Pre-F), varying in different meiotic phases and oogonia/oocytes belonging to different developmental stages were measured for female individuals on different days post-hatch (dph). Information about the cell apoptosis dynamics transition, the changing patterns of maternal 17β-estradiol (E_2_) and endogenous FSH, and the temporal and spatial expression of the FSH receptor and its downstream P450aromatase (CYP19A1) were also determined. Furthermore, an experiment was designed to investigate the dissociated ovarian cell apoptosis response to FSH, E_2_ and estrogen receptor (ESR) agonists in vitro to explore the effect of FSH on pivotal events encompassing oogonial proliferation, the initial germ cell transition from mitosis to meiosis, and Pmf recruitment for further development.

**Methods**

**Animal Tissue and Sample Collection**

Twenty-one *Alligator sinesis* eggs from a single clutch provided by Wuhu Dajiang Farmer (Wuhu, Anhui, China; longitude 118.41°, latitude 31.29°) were collected on the 15^th^ day of incubation prior to the period of sex determination[14]. Based on the experience that 29 ℃ produced 100% females in our previous research[15], all the eggs were carefully transferred to the Alligator Research Center of Anhui Province, set in damp moss and incubated at 29℃ and 90% humidity. The entire incubation period lasted 70 days until the last egg hatched. Based on previous studies focusing on the cause of the formation of compacted yolk in young Chinese alligator[16], newly hatched individuals were given fasting treatment for 30 days. After normal yolk absorption was confirmed, all individuals were given conventional feeding (chicken breast and fish meat mixture (1:1) weighing 8% of body weight). During this period, a subset of 18/21 specimens (one case at 15 dph, 30 dph and 90 dph that did not conform to the typical dimorphic characteristics was excluded) were randomly selected at different days post-hatch (dph), including 1, 15, 30, 60, 90 and 300 dph (N=3 for each dph). After blood plasma (10 ml per individual) collection via the dorsal postcranial sinus, all the individuals were euthanized via decapitation following deep anesthesia with an intraperitoneal injection of sodium pentobarbital (50-100 mg/kg, Propbs, Beijing, China). The cerebrum and both sides of the gonad were rapidly dissected within 5 min of death and further divided into two equal parts for 4% paraformaldehyde fixation and frozen in liquid nitrogen. In addition, the maternal yolk components from the 1- and 15-dph groups were rapidly dissected, weighed, rinsed in freezer ultra-pure water, and placed into 1.5 mL microcentrifuge tubes at -80℃ until further assay.

**Histomorphological observation and counting of germ cell numbers**

After fixation, all the ovarian tissues were deparaffinized and rehydrated through a series of solutions from 100 % to 70% ethanol and deionized water. To perform follicle counting, individual left ovarian tissues were serially sectioned parasagittally at 6 μm and stained with a periodic acid-Schiff staining kit (Beyotime Biotechnology Co., Ltd, Beijing, China) based on the finding that all modern crocodilians have two functional ovaries in gametogenesis and that the number of germ cells did not differ between the two ovaries[16]. To quantify the exact number of germ cells belonging to different developmental stages, the number of oogonia/oocytes within the Nest and Pmf was counted via complete serial section examination under the microscope, and the numbers of oocytes within the PF (19 to 22 μm in diameter) and Pre-VF (52 μm in diameter) were counted every three sections (18 μm) and nine sections (54 μm), respectively. Using this method as a reference, the number of germ cells belonging to different meiosis stages was also determined to calculate the dynamic changes in germ cells within different meiosis processes.

**Maternal yolk and plasma steroid substance analysis**

After thawing, the following steps of yolk isolation for 17β-estradiol (E_2_) extraction were performed according to the protocol described previously[17], with minor modifications. First, all the yolk samples were homogenized into aliquots, topped up to 200 μL with freezer ultra-pure water, and then extracted with 1 mL of diethyl ether by vortex mixing for 1 min. The aqueous and organic phases were allowed to separate for 1 min prior to snap freezing in dry-ice cooled methanol. The yolk E_2_ was measured using a commercial kit (D741007, Sangong, China) as described previously in American alligators[18] with minor modification as following: Firstly, the standard sample and yolk sac extraction were added to the microplate that have been pre-coated with antigen. After incubation, add Biotin-conjugated antibody and then incubated and washed to remove unbound enzyme, and then added to the chromogenic substrate to produce a blue color, and converted to the final yellow under the action of acid. Finally, the absorbance value was measured at 450 nm. The concentration of E_2_ in the yolk sac extraction was proportional to the OD value and calculated by drawing a standard curve. Using this assay, the detection ranged from 1.5 to 800 pg, and sensitivities less than 0.5 pg/g yolk were achieved in the actual measurement. Furthermore, the plasma hormone substances were measured using a commercial ELISA kit for the FSH (D721074, Sangong, China) and E_2_ (D741007, Sangong, China), and the assay range and sensitivities for the measurements were 1.56-100 and 9.37-600 pg/mL and 0.94 and 5.62 pg/mL, respectively. Additionally, the coefficients of the intra-assay and interassay CVs were < 10% and < 15% for the detection between three technical replicates run for each biological replicate.

**Immunohistochemistry**

Following paraformaldehyde fixation and dehydration in a series of graded ethanol concentrations, all paraffin-embedded sections with 6 μm thickness were deparaffinized in xylene and further exposed to citrate antigen retrieval solution in a microwave at 100 ℃ for 15 min to activate the tissue surface antigens. After 1 hour of treatment with immunostaining blocking buffer, the slides of cerebrum and gonad tissues were further incubated overnight at 4 ℃ with primary antibodies against FSH (bs-1536R, Bioss, China, diluted 1:550) and FSHr (bs-20659R, Bioss, China, diluted 1:1000), respectively. Then, all slides were washed twice in PBS and incubated with secondary antibody at a dilution of 1:100 at 37 ℃ for 1 h the next morning. After the secondary antibody was sufficiently washed off with PBS, the sections were colorated and then counterstained with hematoxylin. Images were captured using an electron microscope (Zeiss Imager microscope, Germany).

**TdT-mediated dUTP nick-end labeling (TUNEL) assay for cell apoptosis**

TUNEL assays were performed using a commercial cell kit (C1089, Beyotime, China) to assess cell apoptosis on three random technical replicates from complete serial sections of each biological replicate. According to the manufacturer's instructions, all the selected sections were pretreated with 20 mg/mL proteinase K for 15 min at 37 ℃, washed in PBS and then incubated with the TUNEL reaction mixture for 1 h at 37 ℃. Negative controls were incubated in the absence of the enzyme terminal transferase. After that, the slides were observed under a confocal laser scanning microscope (Zeiss, Germany) with 488-nm excitation and 530-nm emission. The data of the TUNEL-positive percentage of ovarian tissues were examined in a blinded manner via capture and digitization by image analysis software.

**Quantitative real-time PCR**

According to the protocols provided by the manufacturer, total RNAs of ovarian and cerebrum samples were extracted from three independent biological replicates in each group using the RNAprep Pure Tissue Kit (Takara, Dalian, China). The RNA concentration and integrity were quantified by measuring the absorbance at 260 nm using a NanoDrop 8000 spectrophotometer (Thermo Scientific, USA). The RNA integrity was further verified by the presence of sharp bands for 28S and 18S ribosomal RNA through nondenatured agarose gel electrophoresis. The sequences and GenBank accession of the primer sets used for amplifying the reference gene *RPL8*, and the targeted genes such as *FSH* (Follicle stimulating hormone subunit beta), *FSHR* (Follicle stimulating hormone receptor), and premeiotic marker *STRA8* (Stimulated by retinoic acid 8), as well as meiotic markers *SYCP3* (synaptonemal complex protein 3) and *DMC1* (DNA meiotic recombinase 1), are presented in Table 2. The amplification reactions were carried out as follows: 1.6 μl cDNA, 10 μl SYBR green ER master mix, 7.6 μl nuclease-free water, and 0.4 μl each of the forward and reverse primers (10 μmol) were mixed and set at 95 ℃ for 5 min and 41 cycles at 95 ℃ for 5 s and 59 ℃ for 30 s, using a CFX96TM Real-Time PCR Detection System with a SYBR Premix Ex Taq II Kit (TaKaRa, Dalian, China). At the end of the PCRs, relative mRNA quantification was analyzed by using the 2^-△△Ct^ method normalized to the housekeeping gene *RPL8.* The analyses were performed using three technical replicates from each dph group.

**Ovarian cAMP analysis using RIA**

For the RIA analysis, snap frozen ovarian tissues were first solubilized using 100 μl HCl (0.1 M/200 mg). After centrifugation at 12,000 × g for 5 min, the supernatants were collected and then dried overnight at 60°C. According to the manufacturer’s instructions (IM117, Immunotech), a standard curve was constructed using a log-linear curve fit with average counts per minute (cpm) of the paired standards ratio to the cpm of total activity against the cAMP concentration. The ovarian cAMP concentrations were normalized to the amount of ovarian protein measured using the BCA protein assay kit (C503021, Sangong, China). The examination sensitivity was 0.2 nM pg/mL, and the coefficients of intra- and interassay variation were 11% and 16%, respectively. Three biological replicates with three technical replicates were performed.

**Ovarian cell culture and treatment**

Ovarian cells were collected from 30-dph female *A. sinensis* according to the following procedure. In brief, the ovarian tissues were minced using sterilized scissors dissociated with 1 mg/mL collagenase (Gibco, US) in a shaking water bath by 15 min digestion at 37 ℃. After 120-Am cell mesh filtration, the dispersed cell aggregates were cleaned with fresh high glucose DMEM (E600003, Sangong, China) three times by centrifugation at 1200 rpm for 10 min. After cell viability was measured by the trypan blue test (exclusive rate ≥ 90%), all ovarian cells were transferred into a 24-well plate (2.5 ×10^5^ cells per well) with 14 mm round glass coverslips and then randomized into five groups as follows: control group (cultured with high glucose DMEM supplemented with 10 μg/ml insulin, 5 μg/ml transferrin and 30 nM selenite), FSH treatment group (cultured with control medium and treated with 0.5 IU/mL FSH), E_2_ treatment group (cultured with control medium and treated with 10^-3^ mM/mL E_2_), FSH and E_2_ cotreatment group (cultured with control medium and treated with FSH at 0.5 IU/mL and E_2_ at 10^-3^ mM/mL), and DPN treatment group (cultured with control medium accompanied by dose-treatment of DPN at 0.025 mM/ml, 0.1 mM/ml, and 0.25 mM/ml).

All five groups of ovarian cells were cultured at 31 ℃ and 5% CO_2_ for 24 h, and further TUNEL assays were performed using 6 independent biological replicates within six wells obtained from each treatment. Based on the procedure, after washing twice in PBS and diluting to a final volume of 500 μL in PBS, the cell apoptosis of the obtained cells was determined via flow cytometry analysis. Additionally, RT‒PCR analysis for the expression of *scyp3* mRNA was performed using three technical replicates within 6 independent biological replicates from six wells for each group.

**Statistical analysis**

Statistical analyses were performed using the SPSS statistical software program (version 19.0; SPSS Inc., Chicago, IL). The data normality analysis used the Kolmogorov‒Smirnov goodness-of-fit test, and the data that did not follow a normal distribution were analyzed by the χ2 test using Fisher’s exact probability test. Data analysis among different dph groups was performed using one-way ANOVA. Single pairwise comparisons between any two consecutive dph stages were further examined using Tukey’s test. For some relatively quantitative data such as RT-PCR, they were quantified and reported as arbitrary units relative to a set value of 1 for the sample of 1-dph, to describe their changing characteristics in the later stages more intuitively All analyses were performed using three biological replicates from each dph. The corresponding data are presented as the mean ± SEM. The differences were regarded as significant at *P* < 0.05, annotated by the figure columns with different letters and table values with different superscripts.

**Results**

**Developmental morphology characteristics of ovarian tissues**

As demonstrated in Fig. 1A.a, a large number of Nest consisting of tightly packed oogonia with large nuclei and lightly stained ooplasm were observed within the gonad collected at 1 dph. Meanwhile, a small quantity of Pmf, which was characterized as naked primary oocytes attached by one or two squamous pregranulosa cells irregularly scattered between somatic epithelial cells, was also observed. For the gonad collected from 15 dph, although relatively numerous Nest still existed, abundant germ cells inside the Nests displayed enhanced interactions with somatic cells, simultaneous with the notably increasing number of Pmf compared with those in the 1 dph (Fig. 1A.b). At 30 dph (Fig. 1A.c), a large number of Pmf were observed within the medulla stroma that differentiated from the medulla blastema, in which fragmentation regression occurred to form oval or irregularly shaped lacunae compared with ovaries at 1 dph and 15 dph. As shown in Fig. 1A.d and e, some PFs, which were characterized as germ cells surrounded by pyriform-shaped GCs and thecal cells (TCs), started to be observed at 60 and 90 dph. Within the PF, the germ cells showed peripheral nucleoli within the nucleus and contained some lipid globules in the more basophilic cytoplasm. PAS-positive materials developed in the juxtanuclear cytoplasm and consisted of a homogeneous spherical mass of protein and RNA known as the Balbiani body (Ba). In addition, obvious zona pellucida (ZP) could also be observed in the periphery of some primary oocytes within the PFs. Notably, a small number of granulosa cells in the PFs were observed to be transformed from a flat and squamous shape to a pyriform shape (▲, marked in Fig. 1A.e). When developed to 300 dph, a large number of Pre-VFs, which were characterized as oocytes surrounded by obvious layers of GCs and TCs, were observed. For the Pre-VF, the primary oocytes showed lampbrush chromosomes (Lch) and periphery nucleoli and exhibited homogeneous and lightly stained yolk nuclei characterized as small, basophilic, ovoid structures adjacent to the nuclear membrane (Fig. 1A.e).

**Dynamic changes in germ cell number within different follicles**

As shown in Fig. 1B, at 1 dph, the number of germ cells within the nest was approximately 2.3 times greater than that at 15 dph and then declined rapidly at 30 and 60 dph (*P* < 0.05). A second small peak occurred at 90 dph and then dropped to a similar number at 30 and 60 dph (*P* > 0.05). The number of germ cells within the Pmf increased from 1 dph to a peak at 30 dph and then decreased significantly and remained at a low level in the following measurements at 60, 90, and 300 dph (*P* < 0.05). The number of germ cells within the PF was low to undetected at 1, 15 and 30 dph, significantly increased starting from 60 dph, peaked at 90 dph (*P* < 0.05), and further significantly decreased at 300 dph (*P* < 0.05). Pre-VF was not observed in the early developmental stage until 300 dph, at which point appreciable quantities of Nest, Pmf, and PF were still observed, confirming that the processes of oogenesis and folliculogenesis were protracted and asynchronous in crocodilian species.

**Dynamic changes in germ cells with different meiotic stages**

As illustrated in Fig. 2A, the meiosis process of the germ cells within the germ cell nest was mainly stable at leptotene, which contained condensed filamentous chromatin with a large number of concentrated particles, and zygotene, which was characterized by thin and dense fibrous chromatin (Fig. 2A, left panel). In contrast, the meiosis processes of oocytes within the Pmf characterized by the presence of a varying number of GCs (marked with the asterisk) surrounding a single oocyte were observed to show several advances in meiotic stages. For example, a majority of pachytene oocytes experienced fragment exchange between nonsister chromatids, which caused partial recombination nodules. Additionally, a small number of diplotene oocytes contained thick dense fibrous chromatin with evident chiasmas and several peripheral nucleoli located in the decondensed chromatin (Fig. 2A, right panel).

According to the morphological characteristics of the chromosome during different processes of meiotic proliferation, the numbers of oocytes in different meiotic stages were counted (Fig. 2B). Except for the proportion of oocytes in the diplotene stage, which increased linearly with increasing dph (*P*<0.05), there was no significant difference between the 15 dph and 300 dph time steps in the proportion of oocytes belonging to the other meiotic stages, including the leptotene, zygotene, and pachytene stages (*P*>0.05). Additionally, the proportion of oogonia within the premeiosis interphase peaked (approximately 70-80%) at the earliest time step of 1 dph and rapidly decreased to 30-40% during the following developmental stages. The corresponding data demonstrated that the initiation transformation of the oogonia into differentiating oocytes occurred at 15 dph.

**Quantitative real-time PCR analysis of premeiotic and meiotic marker genes**

In order to further understand the developmental patterns of germ cells, the STRA8, SYCP3 and DCM1 were selected as premeiotic and meiotic marker to detected their mRNA abundance changes at different developmental stages. In parallel with the initiation transformation of the oogonia into differentiating oocytes occurred at 15 dph, substantially increased mRNA expression of the premeiotic marker *STRA8,* a specific expression gene in mammalian embryonic ovarian germ cell's transition from mitosis to meiosis*,* were obtained at 15 dph and 30 dph (*P*<0.05). Meanwhile, there was no significant difference in the mRNA expression of the meiotic markers *SYCP3* ( a key component of the synaptonemal complex regulating meiotic homologous recombination) and *DMC1* (an important regulatory factor to generate a homologous chromosomes crossover responsible for segregation of the chromosomes at meiotic division I) among the different dph groups (*P*>0.05).

**Quantification of the TUNEL-positive labeled cells of ovarian tissues at different dph**

As illustrated in Fig. 3A, the TUNEL-labeled apoptotic cells from all the different dph groups did not exceed 10%, and most were located mainly in the medulla region of the ovary. Although significant elevations were detected at both 15 and 30 dph (*P*<0.05), the apoptotic signals in the cortex region, where the specific distribution of germ cells was observed only at 30 dph, indicated the spatial specificity of germ cell degeneration. Moreover, a small amount of TUNEL-positive apoptotic signals were observed in germ cells within the Pre-VF appearing at 300 dph. In parallel, some stromal cells were likewise stained with TUNEL-positive signals.

**Maternal** **17β-estradiol and endogenous FSH**

The endogenous distribution of FSH immunopositive cells was analyzed for the first 3 dph groups (Fig. 4A). Weak positive immunolocalization of FSH was observed as early as 1 dph within the cerebral cortex, including the piriform cortex (PC), neocortex (NC), hippocampus dorsal cortex (HDC), hippocampus inner cortex (HIC) and corpus striatum (CS), as well as in the subcortical centers, such as the dorsal ventricular ridge (DVR), which indicated that the presence of endocrine regulation of the neuroendocrine-gonad axis is an early event during the embryonic period.

As illustrated in Fig. 4C, the concentration of maternal yolk E_2_ at 1 dph ranged from 12.04 to 21.74 picograms/gram of yolk. Nevertheless, the maternal yolk reserves were exhausted when the individual developed to the 15-dph and later development stages (Table 1). Accompanied by the exhaustion of maternal yolk reserves, the plasma E_2_ substantially declined to the lowest level at 15 dph, significantly increased from 30 dph and peaked at 300 dph (*P*<0.05). The greatest increase in plasma FSH concentration occurred at 15 dph, concurrent with the significant increase in FSH immunopositive signals within the cerebrum, such as in the PC, NC, and DVR groups (Fig. 4B). Moreover, the relative expression of *FSH* mRNA relative to the internal control gene (*RPL8*) in cerebrum tissue likewise exhibited a trend of progressive upregulation with increasing dph (Fig. 4C). Taking into account the above results, we postulated that the upregulation of endogenous FSH may be attributed to the weakening of the negative feedback inhibition of maternal steroid hormones.

**Immunostaining of FSHr within follicles at different developmental stages**

As shown in Fig. 5A, the specific immunological localization of FSHr was mainly detected within the germ epithelium but not labeled in the oogonia at 1 dph. Different follicular expression patterns of FSHr were observed between the 15 dph and 60 dph time steps. For instance, the Pmf that showed an oocyte surrounded by one or two squamous pre-GCs at 15 dph and 30 dph was never labeled by anti-FSHR antibodies. Nevertheless, some follicles intermediary between Pmf and PF, those characterized by a larger number of GCs, showed faint immunostaining. Nevertheless, it was difficult to determine whether it was associated with the germ cells or the GCs, as the immunosignal seemed to be present at the boundaries between the oocyte oolemma and the interactively surrounding GCs. At the same time, moderate FSHr in the cortical region, along with immunolabeled undifferentiated somatic cells located within the stroma of the medulla, were simultaneously present at 15, 30, and 60 dph. As observed at 90 dph, the immunolabeled oocytes within the PF were no longer restricted to the areas in contact with the oolemma but were also observed throughout the whole oocyte nucleus. In addition, the unique FSHr-positive signal was present within pyriform cells but absent within the other [squamous](javascript:;) GCs located at the mono-layered GC layer. At 300 dph, the majority of Pre-VFs were labeled with the anti-FSHR antibody. With increasing follicle size, the development of the yolk nucleus permitted the oocyte immunostaining to be distinguished from that of the GCs, which revealed that immunolabeled FSHr signals in the oocytes were more substantial than those in the GCs.

As illustrated in Fig. 5B, the ovarian expression patterns of *FSHr* mRNA showed the lowest levels at 1 and 15 dph and increased significantly with the days post-hatch (*P* < 0.05). Additionally, there was no significant difference in ovarian cAMP among 1, 15, 30, and 60 dph (*P* > 0.05), but ovarian cAMP was significantly elevated from 90 dph and finally peaked at 300 dph (*P* < 0.05, Fig. 5C).

**Immunostaining of CYP19A1 within ovarian tissues**

As illustrated in Fig. 6, the immunofluorescence localization of CYP19A1, a key enzyme encoding aromatase, which is responsible for estrogen biosynthesis, was located only in the medulla region of the 1-dph ovarian. After that, a small amount of CYP19A1 immunoreactivity was occasionally detected in the medulla/cortex interface at 30 dph. Finally, an appreciable number of CYP19A1-positive cells were observed to be incorporated into the putative thecal cell layer of Pre-VF observed at 300 dph.

**Effect of FSH, E_2_ and ESR agonists on dissociated ovarian cell apoptosis in vitro**

To identify the subpopulation of dissociated ovarian cells, the dissociated ovarian cell suspension and coverslips were distinguished by microscopy and immunofluorescence staining for functional marker proteins (Fig. 7A). The corresponding results showed that the ovarian cell suspension obtained after collagenase dissociation included 1) germ cells characterized by conspicuous size, large round nuclei, and spherical Balbiani bodies within the cytoplasm (left panel) and immune-localization staining for the germline marker DDX4 (middle panel). 2) Steroidogenic cells showing large amounts of lipid droplets (left panel) labeled with the steroid hormone synthesis pathway key enzyme 3β-HSD (middle panel). 3) Somatic cells of irregular shape (left panel). To further verify the cell viability, the trypan blue test was performed and the results indicated that the exclusive rate of dissociated ovarian cell suspension was 93% (right panel).

To further explore the mechanism in special follicular assembly characterizing the comparatively lower crocodilian cell apoptosis compared with that of mammals, we further explored the mechanism of cell anti-apoptosis during early gonad development by investigating changes in apoptosis of cultured ovarian fragment cells in response to FSH and E_2_ alone or in combination. The corresponding results indicated that either FSH or E_2_ treatment significantly decreased the number of TUNEL-labeled ovarian apoptotic cells (*P* < 0.05). Together, FSH and E_2_ displayed a reciprocal effect on the inhibition of the ovarian cell apoptotic rate down to 11% compared to the rate of the control group (*P* < 0.05). According to the specificity of the pharmaceutical ESR agonist assay in American alligator, 2,3-bis4-hydroxyphenyl-propionitrile (DPN) has been shown to exhibit comparative specificity for both nuclear estrogen receptors of ESR1 and ESR2, although not displaying the greatest transcriptional specificity [[14](#_ENREF_14)]. Therefore, DNP was selected to investigate the effect of ESR agonists on ovarian cell apoptosis in the present research. The corresponding results indicated that significantly decreased cell apoptosis after exposure to 0.1 mmol/ml DPN, whereas these alterations were not observed after exposure to doses of 0.025 mmol/ml or 0.25 mmol/ml compared to the control group (Figures 7 B and C).

**Discussion**

*The dynamic changes in the number of oocytes indicated that the oogenesis process was asynchronous in crocodilian species*

The oocyte undergoes a dramatic differentiation process that begins when germline stem cells give rise to oogonia, which further divide synchronously with incomplete cytokinesis to form the multinucleated germ cell nest [[2](#_ENREF_2)]. This critical event that occurs during oogenesis has been suggested to be evolutionarily conserved in males and females of species ranging from higher insects to frogs, rodents and other vertebrates [20]. Consistent with the first appearance of meiotic germ cells in stage 24-28 embryos of *Alligator mississippien*sis[21], the presence of a germ cell nest at 1 dph indicated that oogonia mitotic proliferation was initiated before hatching. According to the dynamic changes in the number of oocytes, a small amount of Pmf derived from germ cell nest breakdown appeared at 1 dph, accompanied by an elevated oogonia number at 15 dph, confirming that the oogenesis process was asynchronous in crocodilian species, at least during the process of oogonia mitotic proliferation. This phenomenon was similar to the oogonial division that occurs in birds, in which oogonial mitosis initiates on the embryonic 9^th^ day, and another smaller peak occurs in the chick embryo on day 17[22]. Nevertheless, the particular pattern of lifelong continuous oogonia proliferation in reptiles, including crocodilians, that is inconsistent with the mitosis multiplication that is completed by the time of hatching that has been reported in birds, makes the investigation of the mechanism involved in triggering oogonial proliferation rather complex.

*Endogenous hormonal milieu might be strongly correlated with the initiation of the germ cell meiosis process*

As the first report focusing on the endogenous hormonal milieu during oogonia proliferation, our current data demonstrated that endogenous FSH accompanied by *CYP19A1* mRNA, which is responsible for estrogen biosynthesis, was observed as early as 1 dph. The corresponding results revealed that the presence of endocrine regulation of the pituitary-gonad axis is an early event and may be functional in oogonia division. In the present research, the mitotic activity peak reflecting the significantly increasing number of germ cell nests occurred at 1 dph when FSH levels were observed to be low, which indicated that low levels of FSH might be more conducive to the mitotic proliferation of oogonia during the oogenesis of crocodiles. The above result was not in opposition to the previous findings of reduced oogonial mitotic proliferation after hypophysectomy treatment but explained that the loss of gonadotropin stimulation results in the blocked mitotic proliferation of oogonia. Similarly, oogonial proliferation is augmented during the post-spawning period, when circulating gonadotropin levels are observed to be at the lowest levels in the seasonal breeding cycle of bony fish [23]. On the other hand, several published reports have illustrated that FSH receptors (FSHr) are localized not only in ovarian GCs but also in surface epithelial cells [24]. Our current data indicated that the specific immunological localization of FSHr was observed in the germ epithelium but absent in the oogonia at 1 dph. We further speculated that oogonial mitotic multiplication may not depend on the direct effect of FSH. It also suggests the presence of some intraovarian factors that regulate the heterogeneous characteristics of oogonia mitotic proliferation occurring in the newly hatched *Alligator sinensis*. Direct evidence comes from the finding that the treatment effect of FSH on BrdU incorporation and the mitotic index of germ cells was delayed several hours compared to that seen on the surface epithelium and somatic cells of the ovarian medulla in avians[25].

*Endogenous FSH might be functional in promoting the meiosis onset, sustaining the survival of primordial follicles.*

After the formation of germ cell nests via oogonial mitotic division, the initiation of oogonia transformation into differentiating oocytes in parallel with encapsulation by squamous pre-GCs is crucial for the formation of Pmf through germ cell nest breakdown[26]. It has been proposed that the breakdown of germ cell nests is a coordinated effort that involves the degeneration of many germ cell nuclei and invasion into germ cell nests by pre-GCs [27]. Most of the initial cells within germ cell cysts ultimately develop into a Pmf, with the remaining cells undergoing extensive cell apoptosis in mouse [28], zebrafish [29], and Drosophila [30], with all of these examples suggesting a cell fate decision between apoptosis and follicle formation during germ cell nest breakdown [31]. In contrast, only a few cells in the mitotic cyst or neighboring area undergo cell apoptosis, as reported in Xenopus[32] and Medaka[33]. To our knowledge, information about the transition of cell apoptosis dynamics from germ cell nests to enclosed follicles has not been previously addressed in crocodile species. Our current data illustrated that the TUNEL-labeled apoptotic cells from all the different dph groups were did not exceed 10%. This number was less than those of mammalian species as represented by mice, in which approximately 80% of the vast majority of germ cells cannot escape apoptosis during germ cell development[34, 35]. Excluding the limiting factors caused by TUNEL recognition only for the late apoptosis of cells, we further speculated that these might be associated with the special mechanism in reducing energy loss to maintain the survival of an adequate number of germ cells in the oviparous amniotic species. The above-mentioned postulations provide a reasonable explanation for why it is unnecessary to accommodate such a large stock of germ cells or meiotic oocytes to be used during the reproductive life of mammalian individuals [[5](#_ENREF_5)]. Although the number of germ cells within the germ cell nest peaked in the 15-dph ovarian, the initial sign of breakdown of the germ cell nest, reflecting the advanced abscission stages of the germ cell nest and increased interaction between oocytes and pre-GCs, has also been observed. Meanwhile, the greatest cell apoptotic rate was observed within the 30-dph ovarian tissues, especially in the cortex region, where germ cells are mainly distributed. The results of the current study indicated that germ cell nest breakdown preceded the majority of germ cell loss, and apoptosis was not a major factor in germ cell nest breakdown in crocodile species. Additionally, the undetectable cell apoptotic signal in the cortex region observed at 15 dph also indicated that the decrease in the number of germ cells within the germ cell nest was not due to an increase in atresia resulting from germ cell apoptosis but rather seemed to be attributed to the rising number of oocytes escaping from the germ cell nest and subsequently entering the growing pool.

It has been proposed that the embryonic gonads are not the only source of steroids. The maternal yolk may be a significant source of these substrates and/or estrogen during development in oviparous reptiles [36]. Given the lipid composition of yolk in alligator eggs[37], estrogen and steroid substrates for aromatase synthesis might be stored in the yolk to provide a potential reservoir for maternal steroids [38]. In the present study, with a significant decrease in plasma E_2_ due to the exhaustion of maternal yolk reserves, either the FSH immunopositive signals within the cerebrum or its plasma concentrations both showed a trend of negative feedback augmentation. According to the notably increasing number of Pmf, as well as the advanced meiosis stage reflecting the robust upregulation of the premeiotic marker *STRA8* mRNA observed within 15 dph, we further proposed that endogenous FSH might be functional in promoting the transition of the mitotic division of oogonia to meiosis onset and sustaining the survival of primordial follicles during germ cell breakdown. The corresponding suggestion provided a reasonable explanation for oogonial degeneration, which does appear to be more prevalent in vertebrates that produce a single pool of germ cells during the embryonic period discussed above. Supporting the above speculation, a preliminary investigation reported that estradiol in maternal yolk provides a steroid background on which gonadal development is initiated and proceeds, such as in the abnormal gonadal development of newborn crocodiles[[13](#_ENREF_13)]. However, whether the robust upregulation of endogenous FSH was driven by feedback inhibition of maternal steroid hormones or by pulsatile regulation by upstream hypothalamic GnRH neurons[39] and kisspeptin neurons[40] is still unclear and needs to be further verified in subsequent research. Meanwhile, the synchronization of endogenous FSH upregulation coincided with the occurrence of an increase in the number of leptotene meiotic germ cells, which suggested that neuroendocrine regulation may be strongly correlated with the initiation of the germ cell meiosis process.

Regulation of intercellular bridges between oocytes and surrounding GCs and oocytes diplotene arrest maintenance might be dependent on the action of FSH.

The follicular epithelium undergoes structural and morphological modifications throughout the growth of oocytes. In the reptilia squamata, the undifferentiated somatic cells lining the simple surface epithelium migrate into the ovarian cortex, give rise to prefollicular cells and attach to and invest with primary oocytes to form the Pmf[41]. Seasonal changes in the mitosis phase in surface epithelial cells are suggested to be mediated by gonadotropins and/or gonadal steroids[[11](#_ENREF_11)]. It has been widely acknowledged that FSH plays an important role by binding to FSHR in target ovarian cells to regulate gametogenesis[42]. Although different follicular expression patterns of FSHr were detected, the simultaneous presence of moderate FSHr staining in the cortical region, along with immune-labeled undifferentiated somatic cells located within the medulla stroma, was observed from 15 to 60 dph. According to what the dynamics of changing the oocyte number demonstrated, the increasing number of Pmf at 15 and 30 dph, in parallel with the first appearance of PFs at 60 dph, suggested that FSH likely promotes the proliferation and differentiation of these cells into pre-GCs. This suggestion was consistent with the suggestion that gonadotropins should act on somatic supportive cells and not directly on germ cells either in vivo [43] or in vitro [44]. Additionally, it has been acknowledged that FSH binds only in GCs [45], which led to a consensus that FSH’s action on oocyte development was considered secondary to these effects and was due to diffuse substances, possibly passing through gap junctions between GCs and the oocyte [46]. In the present study, FSHr-positive staining signals were also occasionally observed in the follicles that were intermediary between Pmf and PF, in which the boundaries between the oolemma and the interactively surrounding pre-GCs were faintly stained. In *Podarcis sicu*, the follicle recruitment has been suggested to be under the control of GCs, which was supported by in vitro evidence using germinal beds containing prefollicular stage oocytes cocultured with ovarian follicle cells in the presence/absence of FSH[47]. In conclusion, we further surmised that FSH affects follicle recruitment via the regulation of intercellular bridges between oocytes and surrounding GCs. The direct evidence supporting this conclusion came from the evidence in the current study that the prefollicular oocytes developing inside the germ cell nest were almost stable at the leptotene or zygotene stages. Meanwhile, concurrent with germ cell nest breakdown, oocytes isolated from one another by investing GCs appeared in more advanced meiotic stages, such as the pachytene and diplotene stages.

Oocytes undergo a series of chromosomal changes specific to meiotic prophase during differentiation, and relevant structural modifications have been demonstrated in amphibians [48], fish [49], and mammals [50]. It has been reported that as oocytes develop to the diplotene stage, chromatin characterized by more granulated and vacuolated nucleoli break into several fractions. In the lizard *Pouhrcis sicla*, the nucleolus of small diplotene oocytes appears as a fihrillo-granular structure and is suggested to be related to the extrusion of nuclear proteins or ribosomes into the ooplasm from these organelles[5]. Contrary to the findings in reptiles, our data in the current study implied that the chromatin architecture in PF and Pre-VF showed more or less evenly distributed nucleoli in the periphery of the nucleolus. In addition, as demonstrated in reptile species such as lizards, transferred oocytes contain peculiar cells called pyriform cells[51]. Pyriform cells have been proved to transform from GCs [52] and are involved in ribosome organization. It has been suggested that transformation of GCs into a pyriform shape is preceded by heterologous fusion of the oocyte plasma membrane [53]. Once differentiated, the unique occurrence of pyriform cells in vertebrates is suggested to establish connections between follicle cells and oocytes through intercellular bridges [51, 54]. Histochemical analysis has revealed a nutritive role for the large GCs, explaining the high metabolic rate, nuclear activity and extensive development of microvilli that occurred between oocytes and GCs during the previtellogenic stage of the lizard *Scincus mitranus* [55]. The Balbiani body has been described as an ooplasmic region of high metabolic activity involved in the initial accumulation of various molecules and organelles (e.g., RNAs, mitochondria, Golgi, and lipid bodies)[56]. In the present study, intense FSHr-positive signals were detected in oocytes and differentiated pyriform cells, as well as within the follicles intermediary between PF and Pre-VF. The corresponding results revealed that the effect of FSH might be attributed to the relevant structural modifications that can be related to the rate of ribosome organization, which was reflected in the appearance of a homogeneous spherical mass of protein and RNA known as a Balbiani body observed at 300 dph.

cAMP is suggested to be a well-characterized intracellular second messenger produced by both oocytes and cumulus cells that maintains the meiotic arrest of immature oocytes[57]. It has been demonstrated that the decreasing cAMP concentration in oocytes leads to the resumption of meiosis [58]. FSH has been acknowledged to act in gonadal function mediated by cAMP [45, 59]. The effect of diplotene stage blocking depends on FSHR mRNA in oocytes[60], representing a possible mechanism for the involvement of gonadotrophins in ovarian cAMP regulation[61] and further inhibiting oocyte maturation[62]. In cultured rat GCs, FSH promotes the expression of inhibin through the intracellular second messenger cAMP [63], which has been shown to play a key role in diploid arrest maintenance in the perinatal mouse ovary[64]. The current findings of intensely FSHr-positive labeling observed within the Pre-VF oocytes paralleled the increased cAMP concentration detected within the ovarian tissues collected from 300 dph. The corresponding results indicate that diplotene arrest maintenance during the early vitellogenesis of *Alligator sinensis* may be FSH dependent via increased levels of intraoocyte cAMP.

Preferential selection in asynchronous meiotic initiation may act on somatic supportive cells and not directly on germ cells via regulation of FSH on downstream estrogen

It is worth noting that similar to what is observed in the lineage that evolved from the ancestor of avian species with asynchronous meiotic initiation, meiosis was initiated in some cells, while mitosis was maintained in others[65]. In the present research, oogenesis onset of *Aligator sinensis* was illustrated to be asynchronous, as evidenced by the overlap between the various stages of meiotic prophase throughout the ovarian tissues collected from all days post-hatch. Because the asynchronous trigger of oogonial proliferation and the onset of meiosis seems rather complex, the involvement of its downstream hormone in FSH action on germ cell development should also be considered. It has been reported that aromatase inhibitor cotreatment with FSH inhibited mitosis and accelerated meiosis of germ cells, indicating that estrogens act as a downstream mediator in the action of FSH to promote cell proliferation[25]. In the present study, immune-reactive cells for CYP19A1 that are responsible for estrogen biosynthesis were located only in the medulla at 1 dph and further observed in the medulla/cortex interface when developed to 30 dph in parallel with the significantly increased number of Pmf forming toward the medulla stroma. According to the findings reported for *Alligator mississippiensis*, GCs do not undergo extensive proliferation in the cortex until the follicle moves to the ovarian stroma along the cortex/medulla boundary[[7](#_ENREF_7)]. We further speculated that preferential selection in asynchronous meiotic initiation may act on somatic supportive cells and not directly on germ cells via regulation of FSH on downstream estrogen. In other words, the diffusion gradient of the FSH receptor in parallel with or followed by steroid hormone on GCs may play an important role in the selective rapid growth processes that occur in a certain small number of oocytes and the suppression there in others. In support of this notion, in vitro evidence showed the reciprocal stimulating effect of FSH and E_2_ on accelerated meiotic marker *SYCP3* mRNA expression and inhibited cell apoptosis of ovarian cells.

**Conclusion**

Although various studies in reptiles have provided circumstantial information that FSH has a mediating effect on folliculogenesis, the precise mechanisms of FSH responsible for the entire course of events modulating oogonia proliferation and differentiation were further determined as follows: 1) Oogonial mitosis might not depend on the direct effect of FSH, which also suggests the presence of some intraovarian factors that regulate the heterogeneous characteristics of oogonia mitotic proliferation occurring in the newly hatched *Alligator sinensis*. 2) Pivotal events that transition from mitosis to meiosis of germ cells during nest breakdown may be related to the upregulation of endogenous FSH caused by a weakening of negative feedback inhibition of maternal steroid hormone, which was speculated to be functional in sustaining the survival of follicle cells during the breakdown of the germ cell nest. 3) Oocyte-derived cAMP dependent on endogenous FSH was important for the diplotene arrest maintenance of oocytes during early vitellogenesis. The corresponding results contribute to expanding the understanding of physiological processes and shed some light on common and specific factors responsible for gonadotropin function supporting the notion of early folliculogenesis of crocodilians.

**Declaration**

**Abbreviations**

dph: days post hatched; E_2_: 17β-estradiol; FSH: follicle-stimulating hormone; FSHr: follicle-stimulating hormone receptor; Nest: Germ cell nest; Oog: oogonia; Pmf: primordial follicles; PF: Primary follicle; Pre-F: previtellogenic follicles; PC: Piriform Cortex; NC: Neocortex; HDC: Hippocampus Dorsal Cortex; HIC: Hippocampus Interior Cortex; CS: Corpus Striatum; DVR: dorsal ventricular ridge; GCs: Granulosa cells; TCs: Thecal cells; Ba: Balbiani’s body; ZP: Zona pellucida; CYP19A1: P450aromatase; PAS: Periodic acid-Schiff; RIA: Radioimmunoassay; TUNEL: TdT-mediated dUTP Nick-End Labeling; RT-qPCR: Reverse transcription-quantitative polymerase chain reaction; *STRA8*: Stimulated by retinoic acid 8; *SYCP3*: synaptonemal complex protein 3; *DMC1*: DNA meiotic recombinase 1; DMEM: Dulbecco's modified eagle medium; DPN: 2,3-bis4-hydroxyphenyl-propionitrile; NCBI: National Center for Biotechnology Information; bp: Base pair; SE: Standard error; *P*: *P*-value.

**Ethics approval and consent to participate**

This project specifically approved by Ethics Committee of Anhui Normal University(AHNU-ET2021008). The animal protocols used in this study were in accordance with Measures for the Administration of the Permit for experimental animals provided by Ministry of Science and Technology of the People's Republic of China (2nd ed. No. 593, 2001).

**Consent for publication**

Not applicable.

**Availability of data and material**

The data that support will be shared upon reasonable request to the corresponding author.

**Competing interests**

All authors declare that they have no conflict of interests.

**Funding**

This work was supported financially by the this research was partially supported by National Natural Science Foundation (31872253), Natural Science Foundation of Anhui Province (2008085QC108), and Major science and technology project of Anhui Province (202003a06020068).

**Authors’ contributions**

HTN designed the study and completed most of an early draft of the manuscript, YLX, YQZ and YX conducted immunohistochemistry, TUNEL assay for cell apoptosis and quantitative real-time PCR, YW and XZJ conducted PAS staining and germ cell number counting, maternal Yolk and Plasma steroid hormones concentrations analysis, and ovarian cells culture and treatment; YKZ and RPW responsible for Artificial hatching and sex identification of crocodile eggs. XBW led the team and revised the analyses and completed the final manuscript. All authors read and approved the final manuscript.

**Acknowledgements**

We thank SQ Sun, S Zhong, TS Xia, JS Nie (Alligator Research Center of Anhui Province) for the Incubation facilities support.

**References**

1. Pepling ME, Spradling AC. Female mouse germ cells form synchronously dividing cysts. Development. 1998; 125:3323-3328.

2. Candace Tingen, Kimteresa A, Woodruff K. The primordial pool of follicles and nest breakdown in mammalian ovaries. Mol Hum Reprod. 2009; 15:795-803.

3. Bristol-Gould SK, Kreeger PK, Selkirk CG, Kilen SM, Mayo KE, Shea LD, Woodruff TK. Fate of the initial follicle pool: empirical and mathematical evidence supporting its sufficiency for adult fertility. Dev Biol. 2006; 298:149-154.

4. Calderón M, Pérez G, Pinilla M. Morphology of the ovary of Caiman crocodilus (Crocodylia: Alligatoridae). Ann Anat. 2004; 186:13-24.

5. Guraya SS. Ovarian Follicles in Reptiles and Birds. Zoophysiology. 1989; 24:1-283.

6. Joss J. Gonadal development and differentiation in Alligator mississippiensis at male and female producing incubation temperatures. Proc Zool Soc Lond. 2010; 218:679-687.

7. Moore B, Uribe-Aranzábal M, Boggs A, Guillette LJ. Developmental morphology of the neonatal alligator (Alligator mississippiensis) ovary. J Morphol. 2010; 269:302-312.

8. Moore B, Hamlin HJ, Botteri NL, Lawler AN, Mathavan KK, Guillette LJ. Posthatching development of Alligator mississippiensis ovary and testis. J Morphol. 2010; 271:580-595.

9. Uribe MCA, Guillette LJ. Oogenesis and ovarian histology of the American alligator Alligator mississippiensis. J Morphol. 2000; 245:225-240.

10. Risley PL. A comparison of effects of gonadotropic and sex hormones on the urogenital systems of juvenile terrapins. J Exp Zool Part A. 1941; 87:435-441.

11. Tokarz RR. An autoradiographic study of the effects of mammalian gonadotropins (follicle-stimulating hormone and luteinizing hormone) and estradiol-17beta on [3H]thymidine labeling of surface epithelial cells, prefollicular cells, and oogonia in the ovary of the liza. Gen Comp Endocrinol. 1978; 35:179-188.

12. Forbes TR. Studies on the reproductive system of the alligator. 1. The effects of prolonged injections of pituitary whole gland extract in the immature alligator. Anat Rec. 1937; 70:113-137.

13. Li HE. Influence of temperature on egg incubation of the wild chinese alligator (alligator sinensis). Acta Zool Sin. 2002; 420-424.

14. Wang RP. Preliminary Analyses on Relationship between Body Temperature of Chinese Alligator and Its Environmental Temperature. Chin J Zool. 2008;15-27.

15. Johnson AL. Reproduction in the Female. Springer New York. 1986.

16. Hamlin HJ, Lowers RH, Albergotti LC, Mccoy MW, Mutz J, Guillette LJ. Environmental influence on yolk steroids in American alligators (Alligator mississippiensis). Biol Reprod. 2010; 83:736-741.

17. Lance VA, Rostal DC, Elsey RM, Iii P. Ultrasonography of reproductive structures and hormonal correlates of follicular development in female American alligators, Alligator mississippiensis, in southwest Louisiana. Gen Comp Endocrinol. 2009; 162:251-256.

18. Hamlin HJ, Lowers RH, Albergotti LC, Mccoy MW, Mutz J, Guillette LJ. Environmental influence on yolk steroids in American alligators (Alligator mississippiensis). Biol Reprod. 2010; 83:736-741.

19. Satomi K, Bernhard MC, Yoshinao K, Jianguo Z, Bryan TA, Doheny BM, Taisen I, Guillette LJ. Estrogen receptor 1 (ESR1; ERα), not ESR2 (ERβ), modulates estrogen-induced sex reversal in the American alligator, a species with temperature-dependent sex determination. Endocrinology. 2018; 2015:1887-1899.

20. Pepling ME, Cuevas MD, Spradling AC. Germline cysts: a conserved phase of germ cell development? Trends Cell Biol. 1999; 9:257-262.

21. Smith CA, Joss J. Gonadal sex differentiation in Alligator mississippiensis, a species with temperature-dependent sex determination. Cell Tissue Res. 1993; 273:149-162.

22. Hughes GC. THE POPULATION OF GERM CELLS IN THE DEVELOPING FEMALE CHICK. J Embryol Exp Morphol. 1963; 11:513-528.

23. Ball JN. Reproduction in female bony fishes. 1960; 178-179.

24. Parrott JA, Doraiswamy V, Kim G, Mosher R, Skinner MK. Expression and actions of both the follicle stimulating hormone receptor and the luteinizing hormone receptor in normal ovarian surface epithelium and ovarian cancer. Mol Cell Endocrinol. 2001; 172:213-222.

25. He B, Mi Y, Zhang C. Gonadotropins regulate ovarian germ cell mitosis/meiosis decision in the embryonic chicken. Mol Cell Endocrinol. 2013; 370:32-41.

26. Elkouby YM, Mullins MC. Coordination of cellular differentiation, polarity, mitosis and meiosis – New findings from early vertebrate oogenesis. Dev Biol. 2017; 430:275-287.

27. Pepling ME, Spradling AC. Mouse ovarian germ cell cysts undergo programmed breakdown to form primordial follicles. Dev Biol. 2001; 234:339-351.

28. Bolcun-Filas E, Rinaldi VD, White ME, Schimenti JC. Reversal of Female Infertility by Chk2 Ablation Reveals the Oocyte DNA Damage Checkpoint Pathway. Science. 2014; 343:533-536.

29. Bontems F, Stein A, Marlow F, Lyautey J, Gupta T, Mullins MC, Dosch R. Bucky Ball Organizes Germ Plasm Assembly in Zebrafish. Current Biology Cb. 2009; 19:414-422.

30. Cathleen, M., Lake, R., Scott, Hawley. The Molecular Control of Meiotic Chromosomal Behavior: Events in Early Meiotic Prophase in Drosophila Oocytes. Annu Rev Physiol. 2012; 74:425-451.

31. Candace Tingen AKKW. The primordial pool of follicles and nest breakdown in mammalian ovaries. Mol Hum Reprod. 2009; 15:795-803.

32. Kloc M, Bilinski S, Dougherty MT, Brey EM, LD E. Formation, architecture and polarity of female germline cyst in Xenopus. Development Biology. 2004; 266:43-61.

33. Nakamura, Shuhei, Kobayashi, Kayo, Nishimura, Toshiya, Higashijima, Shin-ichi, Tanaka, Minoru. Identification of Germline Stem Cells in the Ovary of the Teleost Medaka. Science. 2010; 328:1561-1563.

34. Bullejos M, Koopman P. Germ cells enter meiosis in a rostro-caudal wave during development of the mouse ovary. Mol Reprod Dev. 2010; 68:422-428.

35. Ignacio B, Rodríguez A, Pariente JA. Apoptosis Is a Demanding Selective Tool During the Development of Fetal Male Germ Cells. Front Cell Dev Biol. 2018; 6:65.

36. Groothuis T, Hsu BY, Kumar N, Tschirren B. Revisiting mechanisms and functions of prenatal hormone-mediated maternal effects using avian species as a model. Philosophical Transactions of The Royal Society B Biological Sciences. 2019; 374:20180115.

37. Noble RC, Deeming DC, Ferguson M, Mccartney R. The effect of incubation temperature on yolk lipid parameters during embryonic development of the alligator (Alligator mississippiensis). Lipids. 1993; 28:135-140.

38. Conley AJ, Elf P, Corbin CJ, Dubowsky S, Fivizzani A, Lang JW. Yolk Steroids Decline during Sexual Differentiation in the Alligator. Gen Comp Endocrinol. 1997; 107:191-200.

39. Nie H, Zhang R, Yu X, Zhang Y, Yan P, Li E, Wang R, Wu X. Molecular cloning, immunological characterization, and expression analysis of gonadotropin-releasing hormone (GnRH) in the brain of the Chinese alligator during different stages of reproductive cycle. Gene. 2022; 789:145672.

40. Zhang R, Nie H, Duan S, Yan P, Izaz A, Wang R, Zhou Y, Wu X. Cloning, characterisation and expression profile of kisspeptin1 and the kisspeptin1 receptor in the hypothalamic-pituitary-ovarian axis of Chinese alligator Alligator sinensis during the reproductive cycle. Reprod Fertil Dev. 2020; 32:792–804.

41. Varma SK. Morphology of ovarian changes in the garden lizard, Calotes versicolor. J Morphol. 1970; 131:195-209.

42. Casarini L, Crépieux P. Molecular Mechanisms of Action of FSH. Front Endocrinol. 2019; 10:305-315.

43. Oktay K, Newton H, Mullan J, Gosden RG. Development of human primordial follicles to antral stages in SCID/hpg mice stimulated with follicle stimulating hormone. Hum Reprod. 1998:1133-1138.

44. Cortvrindt R, Smitz J, Van S. Assessment of the need for follicle stimulating hormone in early preantral mouse follicle culture in vitro. Hum Reprod. 1997:759-768.

45. Manuela S, Jörg G, Eberhard N. The follicle-stimulating hormone receptor: biochemistry, molecular biology, physiology, and pathophysiology. Endocr Rev. 1997:739-773.

46. Magoffin D, Kumar A, Yildiz B, Azziz R: Endocrinology of the Ovary. Endocrinology of the Ovary; 2005.

47. Sica S, Fierro G, Iodice C, Muoio R, Filosa S, Motta CM. Control of oocyte recruitment: Regulative role of follicle cells through the release of a diffusible factor. Mol Reprod Dev. 2001.

48. Gansen PV, Schram A. Evolution of the nucleoli during oogenesis in Xenopus laevis studied by electron microscopy. J Cell Sci. 1972; 10:339.

49. Raikova EV. Evolution of the nucleolar apparatus during oogenesis in Acipenseridae. J Embryol Exp Morphol. 1976; 35:667.

50. Antoine N, Lepoint A, Baeckeland E, Goessens G. Evolution of the rat oocyte nucleolus during follicular growth. Biol Cell. 2012; 59:107-112.

51. Klosterman LL. Ultrastructural and quantitative dynamics of the granulosa of ovarian follicles of the lizard Gerrhonotus coeruleus (Family Anguidae). J Morphol. 1987; 192:125–144.

52. Filosa S, Tadde C, Andreuccetti P. The differentiation and proliferation of follicle cells during oocyte growth in Lacerta sicula. J Embryol Exp Morphol. 1979; 54:5-15.

53. Andreuccetti P, Taddei C, Filosa S. Intercellular bridges between follicle cells and oocyte during the differentiation of follicular epithelium in Lacerta sicula Raf. J Cell Sci. 1978; 33:341.

54. Neaves WB. Intercellular bridges between follicle cells and oocyte in the lizard, Anolis carolinensis. The Anatomical Record. 2010; 170:285-301.

55. Aldokhi OA, Alwasel S, Harrath AH. Ultrastructural and histochemical study of previtellogenic oogenesis in the desert lizard Scincus mitranus (Squamata, Sauropsida). J Morphol. 2019.

56. Guraya SS. Recent Advances in the Morphology, Cytochemistry, and Function of Balbiani's Vitelline Body in Animal Oocytes. International Review of Cytology-a Survey of Cell Biology. 1979; 59:249-321.

57. Camp TA, Rahal JO, Mayo KE. Cellular Localization and Hormonal Regulation of Follicle-Stimulating Hormone and Luteinizing Hormone Receptor Messenger RNAs in the Rat Ovary. Mol Endocrinol. 1991; 5:1405-1417.

58. Conti M, Hsieh M, Zamah AM, Oh JS. Novel signaling mechanisms in the ovary during oocyte maturation and ovulation. Mol Cell Endocrinol. 2012; 356:65-73.

59. Zhang M, Xia G. Hormonal control of mammalian oocyte meiosis at diplotene stage. Cell Mol Life Sci. 2012; 69:1279-1288.

60. Patsoula E. Expression of mRNA for the LH and FSH receptors in mouse oocytes and preimplantation embryos. Reproduction (Cambridge, England). 2001; 121:455-461.

61. Marsh JM, Mills TM, Lemaire WJ. Cyclic AMP synthesis in rabbit graafian follicles and the effect of luteinizing hormone. Biochim Biophys Acta. 1972; 273:389-394.

62. Hillensj T, Lemaire WJ. Gonadotropin releasing hormone agonists stimulate meiotic maturation of follicle-enclosed rat oocytes in vitro. Nature. 2019; 1980(287),5778:145-146.

63. Zhang Z, Carson RS, Herington AC, Lee VWK, Burger HG. Follicle-stimulating hormone and somatomedin-C stimulate inhibin production by rat granulosa cells in vitro. Endocrinology. 1987; 120:1633-1638.

64. Wang Y, Teng Z, Li G, Mu X, Wang Z, Feng L, Niu W, Huang K, Xiang X, Wang C. Cyclic AMP in oocytes controls meiotic prophase I and primordial folliculogenesis in the perinatal mouse ovary. Development. 2015; 142:343-351.

65. Smith CA, Roeszler KN, Bowles J, Koopman P, Sinclair AH. Onset of meiosis in the chicken embryo; evidence of a role for retinoic acid. BMC Dev Biol. 2008; 8:85-93.
